# Workplace digitalization and workload: changes and reciprocal relations across 3 years

**DOI:** 10.1038/s41598-024-56537-w

**Published:** 2024-03-11

**Authors:** Hannes Zacher, Cort W. Rudolph

**Affiliations:** 1 Wilhelm Wundt Institute of Psychology, Leipzig University, Neumarkt 9-19, 04109 Leipzig, Germany; 2 Department of Psychology, Wayne State University, Detroit, USA

**Keywords:** Digitalization, Information communication technology, Workload, Longitudinal study, Human behaviour, Psychology

## Abstract

This article reports the results of a 33-wave longitudinal study of changes in, and reciprocal relations between, workplace digitalization and workload. Monthly data were collected between April 2020 and December 2022 from *n* = 1661 employees in Germany. Based on theoretical models of workplace information and communication technology use, stress, and coping, we hypothesized both positive and negative within-person effects of digitalization on workload, and vice versa. Results of an autoregressive latent trajectory model with structured residuals (ALT-SR) showed on-average positive linear trajectories in digitalization, but not in workload over time. Moreover, higher digitalization was associated with subsequently higher levels of workload, and vice versa. This pattern of results suggests a dynamic, reciprocal process wherein positive deviations from one’s average trajectory of digitalization (workload) are associated with subsequently higher levels of workload (digitalization). We additionally find evidence for linear trends in these within-person processes, suggesting that the strength of the within-person effects of digitalization on workload, and of workload on digitalization, becomes more strongly positive over time. Practitioners developing work design interventions could focus on ways to reduce the detrimental impact of digitalization on increased workload, while simultaneously encouraging the potential of digitalization to help employees cope effectively with their workload.

## Introduction

Digitalization, which is defined as “… the way many domains of social life are restructured around digital communication and media infrastructures.…”^1^, p. 556, is a ubiquitous feature of contemporary societies and, consequently, modern work environments^2^. In the workplace, digitalization often involves the use of, and dependency on, information and communication technology (ICT) to successfully carry out one’s work tasks^3^. Workplace digitalization can be a double-edged sword, with both positive (e.g., increased perceptions of productivity^4^) and negative (e.g., increased exhaustion^5^) implications for employees and organizations (see^6^, for a review). For example, digitalization can facilitate communication between coworkers, but can also lead to feelings that one cannot “disconnect” after normal working hours^7^. Arguably, the COVID-19 pandemic accelerated various already present features of workplace digitalization, as employees were called on to rapidly adapt to new technologies and associated modes of working to help curb the spread of the virus (e.g., working remotely and virtually is, to a large extent, facilitated by technology^8^).

A large body of research has demonstrated both positive and negative effects of workplace digitalization on individual-level employee outcomes, such as work performance and job strain^6^. In contrast, the effects of digitalization on various important aspects of work design have received relatively little attention in the literature. Work design refers to “…the content and organization of one’s work tasks, activities, relationships, and responsibilities…”^9^, p. 662. A limitation of research on digitalization and work design is that empirical studies have confounded workplace digitalization (e.g., ICT use intensity) with other work characteristics, such as employees’ workload. For example, research has investigated composite constructs such as “ICT demands”, “work overload due to office-home smartphone use”, “e-mail stressors overload”, or “workplace telepressure” that confound digitalization and its potential work-related antecedents or consequences^10^. Moreover, to our knowledge, no research in this domain has used a longitudinal research design with multiple measurement waves, which allows for testing theoretically-plausible within-person and reciprocal effects among digitalization and various aspects of work design^11,12^. To this end, a recently proposed theoretical model of workplace ICT use suggests that digitalization affects four key work characteristics, namely job demands (i.e., workload), job autonomy, relational aspects of work, and job significance^6^. This model conceives digitalization as an antecedent only, however, and does not consider whether and how (i.e., positively or negatively) work design may also have reverse effects on digitalization.

Accordingly, the goals of our study are threefold: First, based on theoretical linkages drawn from the model of workplace ICT use^6^, we test whether digitalization leads to general increases or decreases in workload at the within-person level of analysis. *Workload* is a key work characteristic that represents the perceived amount and difficulty of work in terms of the volume and pace of work required of an employee^13,14^. Second, we extend the model of workplace ICT use by additionally investigating the plausible reverse effect of workload on digitalization. On the one hand, based on theoretical models of stress and coping^15^, workload could lead to increases in digitalization at the within-person level of analysis. In particular, when employees appraise high levels of workload as a challenge that can be addressed, digitalization may represent a way for them to cope successfully with such high workload. On the other hand, when employees appraise high workload as a threat that is unlikely to be addressed successfully, efforts to digitalize workplaces and make use of technology may be reduced. Finally, we consider several exploratory analyses to examine how both digitalization and workload change over time and how the strength of their associations with one-another changes over time. We accomplish these goals by making use of longitudinal survey data provided by 1661 employees in Germany, who participated in a study with 33 monthly surveys collected between April 2020 and December 2022. We test our competing hypotheses in an integrative framework, using an autoregressive latent trajectory model with structured residuals (ALT-SR^16^).

Our study contributes to the literatures on digitalization and work design in at least three important ways. First, we contribute to a growing knowledge base regarding workplace digitalization^2,3^ by examining its effects on workload as a key work characteristic. Based on a theoretical model of workplace ICT use^6^ and research concerning associations between digitalization and work demands, we propose competing hypotheses on the effects of digitalization on workload at the within-person level of analysis. Importantly, we model such dynamic effects while simultaneously accounting for between-person differences in such variables, ruling out alternative explanations for our findings. Second, based on theoretical models of stress and coping^15^, we contribute to research on workload^14,17^ by investigating whether workload has a generally positive or negative effect on workplace digitalization over time. Research has investigated associations between workload and other work characteristics, such as social support, and employee outcomes, such as strain, but has not considered digitalization as an outcome^18^. An overarching contribution of our study is that we do not confound digitalization and workload as a composite construct, such as “ICT demands”, but examine them as distinct constructs over time. Finally, most research on workplace digitalization has used cross-sectional research designs or longitudinal designs with relatively few measurement waves, which limits conclusions that can be drawn about long-term within-person changes in this variable. We advance the literature by applying state-of-the-art statistical modeling techniques to a large, representative dataset collected at 33 monthly measurement waves to model long-term changes in digitalization, workload, the reciprocal associations between digitalization and workload, as well as changes in strength of these relations over time.

### Effects of workplace digitalization on workload

According to theorizing on workplace ICT use^6^, digitalization may lead to either increases or decreases in workload over time. On the one hand, high levels of workplace digitalization may bring new job demands, such as increased information processing demands and information overload, as well as novel learning requirements and ICT-related technical problems, thus potentially increasing employees’ workload in unintended ways. On the other hand, workplace digitalization involves introducing largely supportive tools (e.g., computers, emails, chat software) that can help employees carry out their work tasks with less cognitive effort and, thus, should lead to reductions in workload^19^. Consistently, a review of 23 papers, which mostly included studies focusing on the between-person level of analysis, found mixed support for the association between workplace digitalization and workload^6^. For example, digitalization has been shown to be associated with more ICT-enabled multitasking^20^, continuous ICT-related learning and adaptation requirements^21^, and ICT-related threats, hassles, malfunctions, and interruptions^22,23^. These ICT-related demands and problems are likely to lead to increased workload. In contrast, other studies have shown that digitalization may help employees save cognitive effort and energy^24^, as it enables them to work more effectively, especially on non-routine tasks^4^. Accordingly, digitalization could also lead to reduced workload over time. Overall, based on theorizing on workplace ICT use and related empirical research, we propose two competing hypotheses regarding potential within-person effects of workplace digitalization on workload:

#### Hypothesis 1

Workplace digitalization leads to an *increase* in workload.

#### Hypothesis 2

Workplace digitalization leads to a *decrease* in workload.

### Effects of workload on workplace digitalization

Theorizing on workplace ICT use has considered only the effect of workplace digitalization on workload and neglected the plausible reverse effect of workload on digitalization^6^. However, it is also likely that the experience of high levels of workload, over time, lead to increases or decreases in digitalization. Theorizing on stress and coping^15^ suggests that work-related stressors and demands, such as high workload, are appraised in one of two different ways. On the one hand, a high workload may be appraised by employees as a challenge (i.e., a situation that, when addressed successfully, offers the potential for growth, mastery, and gain). Work-related challenges can be addressed successfully through problem-focused coping. Increasing workplace digitalization represents a form of problem-focused coping, because digitalization, in its various forms, can help employees carry out certain aspects of their work more effectively, with less cognitive effort^24^. On the other hand, a high workload may also be appraised by employees as a threat (i.e., a situation that cannot easily be addressed and that is perceived to potentially result in harm or loss). Threats are unlikely to be resolved successfully and, thus, require internal changes, such as goal disengagement or emotional coping to manage. When a high workload is perceived as a threat, it is less likely that digitalization, as an aspect of work design, is used to cope with the situation. Indeed, when experiencing high workload, it is even likely that digitalization declines because employees focus more on themselves rather than on proactively changing the design of their work through digitalization. For instance, employees may be more inclined to use emotional coping strategies, such as goal disengagement or self-blame^25^ in such situations. They may also “fall back” on “tried and true” approaches to addressing their workload, rather than on novel technologies. Based on these theoretical considerations, we propose the following two competing hypotheses regarding potential within-person effects of workload on workplace digitalization:

#### Hypothesis 3

Workload leads to an *increase* in workplace digitalization.

#### Hypothesis 4

Workload leads to a *decrease* in workplace digitalization.

## Methods

### Transparency and openness

Data, R code to replicate the analyses, and complete results are available in our Supplemental Materials: https://osf.io/5gebw/. The data used in this paper were taken from a larger longitudinal study that started in December 2019 (Time [T] 1), and included monthly data collections between March 2020 (T2) and December 2022 (T35). Notably, measures of workplace digitalization and workload were only collected simultaneously from April 2020 (T3) on, so our focus here is on 33 monthly measurement waves for which data on workplace digitalization and workload are available. Several articles based on the same dataset have so-far been published (see Table S1 in the Supplemental Materials for a data transparency matrix). These previous articles have completely different research questions and include different substantive variables than the current study. Workload data collected at five measurement waves (i.e., T3, T4, T6, T7, T8) were included in a previous study on a different topic (i.e., family demands and family satisfaction) as an exploratory control variable^26^.

### Study design, participants, and procedure

Workplace digitalization and workload were measured 33 times, first at the beginning of April 2020 (T3), then monthly thereafter until December 2022 (T35). Additionally, demographic characteristics (i.e., age, sex) and socioeconomic status (i.e., education, income, industry) were assessed in December 2019 (T1) and again in November 2021 (T22; to collect additional demographics from two refresher samples added at T6 and T9). To ensure the quality of survey data, an ISO 26362 certified professional panel company was commissioned to recruit participants from a nationally representative online panel in Germany. To qualify, participants had to be at least 18 years old and be working full-time. The panel of *n* = 1661 considered here represents participants who provided data on workplace digitalization and workload at least three times (i.e., the minimum number of data points to model longitudinal change^12^) across the 33 possible waves of this study (see Supplemental Materials for sample descriptive statistics).

### Measures

Reliabilities are reported as average and ranges across time points for which measures were collected (see Supplemental Materials for complete reliability results).

#### Workplace digitalization

At all 33 time points, digitalization was assessed with a 5-item scale from a “Work 4.0” instrument^3^. The items are: “In the last 4 weeks…” (1) “…I was dependent on digital media (e.g., internet, email) to complete my work tasks”, (2) “…I used technical equipment (e.g., smartphone, laptop, computer) provided by my employer to complete my work tasks”, (3) “…I often used information technology (e.g., specific software) during my work”, (4) “…I used information technology in my everyday work, which requires specific training”, and (5) “…without the use of information technology (e.g., internet, software), it would have been impossible to carry out my work.” Responses to each item were collected on a 7-point scale ranging from 1 = *not true at all* to 7 = *completely true*. Average reliabilities observed over time were acceptable (⍺_mean_ = 0.926, ⍺_range_ = 0.887–0.948; ⍵_mean_ = 0.941 ⍵_range_ = 0.906–0.961).

#### Workload

At all 33 time points, workload was assessed with three items from a quantitative workload inventory^13^: “In the last 4 weeks…” (1) “…How often did your job require you to work very fast?” (2) “…How often did your job require you to work very hard?” and (3) “…How often was there a great deal to be done at work?” Responses to each item were collected on a 7-point scale ranging from 1 = *never* to 7 = *always*. Average reliabilities observed over time were acceptable (⍺_mean_ = 0.910, ⍺_range_ = 0.874–0.933; ⍵_mean_ = 0.913 ⍵_range_ = 0.878–0.935).

#### Demographic and socioeconomic status characteristics

Demographics (i.e., age, sex) and indicators of socioeconomic status (i.e., education, income, industry) were collected from respondents at both T1 and T22 (n.b., the latter referring to refresher samples; see above). Age was represented as time since birth (i.e., in years), and sex was represented as a dichotomous variable (i.e., “dummy” coded as 0 = *male*, 1 = *female*). Education was operationalized in terms of highest educational attainment, represented by an ordered categorical variable ranging from 0 = *lower secondary school* to 3 = *college/university or technical school*. Monthly household income was represented as an ordered categorical variable ranging from 0 = €0–€999 to 7 = €6000 to €6999. Finally, industry was operationalized as a dichotomous variable (0 = *primary and secondary sector*, 1 = *tertiary sector*). Primary and secondary sectors refer to industries involved in the extraction of raw materials and manufacturing, whereas the tertiary sector refers to service industries. We report these demographics and indicators of socioeconomic status as descriptive statistics in Table 1 to characterize our sample.

**Table 1:** Sample demographics and comparisons of complete and incomplete responders.

	T3–T35 incomplete	T3–T35 complete	p-value	T3–T35 panel
	(N = 1513)	(N = 148)		(N = 1661)
Age (years)				
Mean (SD)	46.0 (11.2)	46.3 (9.24)	0.72	46.0 (11.0)
Median [min, max]	47.0 [18.0, 71.0]	47.0 [23.0, 69.0]		47.0 [18.0, 71.0]
Missing	1 (0.1%)	0 (0%)		1 (0.1%)
Sex				
Male	831 (54.9%)	93 (62.8%)	0.08	924 (55.6%)
Female	680 (44.9%)	55 (37.2%)		735 (44.3%)
Missing	2 (0.1%)	0 (0%)		2 (0.1%)
Education				
Lower Secondary School	220 (14.5%)	22 (14.9%)	0.33	242 (14.6%)
Intermediate Secondary School	340 (22.5%)	23 (15.5%)		363 (21.9%)
Upper Secondary School	346 (22.9%)	33 (22.3%)		379 (22.8%)
College/University or Technical College	269 (17.8%)	35 (23.6%)		304 (18.3%)
Missing	132 (8.7%)	16 (10.8%)		148 (8.9%)
Monthly household income (euros/month)	48 (3.2%)	7 (4.7%)		55 (3.3%)
€0–€999	93 (6.1%)	10 (6.8%)		103 (6.2%)
€1000–€1999	65 (4.3%)	2 (1.4%)		67 (4.0%)
€2000–€2999				
€3000–€3999	104 (6.9%)	2 (1.4%)	0.08	106 (6.4%)
€4000–€4999	537 (35.5%)	54 (36.5%)		591 (35.6%)
€5000–€5999	263 (17.4%)	28 (18.9%)		291 (17.5%)
€6000–€6999	595 (39.3%)	62 (41.9%)		657 (39.6%)
Missing	14 (0.9%)	2 (1.4%)		16 (1.0%)
Industry				
Primary and secondary sector	179 (11.8%)	18 (12.2%)	1.00	197 (11.9%)
Tertiary sector	1298 (85.8%)	130 (87.8%)		1428 (86.0%)
Missing	36 (2.4%)	0 (0%)		36 (2.2%)

### Analytical strategy

We used an autoregressive latent trajectory model with structured residuals (ALT-SR^16^), which has also been called a latent curve model with structured residuals (LCM-SR^27^), to test our competing hypotheses. The model was specified in a structural equation modeling framework using the R package lavaan^28^ with a maximum likelihood (ML) estimator. The ALT-SR model combines aspects of several traditional approaches to modeling over-time changes, including cross-lagged panel models (CLPMs) and latent growth models (LGMs). ALT-SR models extend the usefulness of these traditional approaches by allowing for simultaneously modeling within-person cross-lagged relations between subsequent time points (i.e., T_*k*_ digitalization predicting T_*k*+1_ workload; T_*k*_ workload predicting T_*k*+1_ digitalization), while accounting for between-person levels (i.e., average, person-level relations between digitalization and workload) and slopes (i.e., average trajectories of change over the entire study period) associated with these variables. Traditional CLPMs confound between-person and within-person variance, which leads to equivocal conclusions about the level (i.e., between-person “average” effects vs. within-person “dynamic” effects) at which modeled relations occur^29^. The ALT-SR model addresses this issue by separating between-person differences and within-person processes from one-another. The model also affords a great deal of flexibility in the parametrization of change over time (e.g., modeling forms of change in within-person cross-lagged effects over time through various model constraints, described below). The ALT-SR model also allows for average, between-person time-graded trends to be modeled and accounted for (i.e., “trajectories” in traditional LGMs), while simultaneously considering within-person processes.

Importantly, the interpretation of cross-lagged effects in ALT-SR models should be considered in some detail, as they diverge from traditional CLPMs. Specifically, positive or negative observed cross-lagged effects (i.e., reflecting relatively “higher” or “lower” over time processes, respectively) must be interpreted in terms of deviations from an individual’s average (i.e., overall) trajectory. Specifically, a positive cross-lagged effect refers to time periods when an individual’s levels are higher than their average trajectory; whereas a negative cross-lagged effect refers to time periods when an individual’s levels are lower than their average trajectory. All parameter estimates for over-time effects described here are in a raw (i.e., unstandardized) metric; however, we provide complete results of standardized parameter estimates in our Supplemental Materials.

**Figure 1:**
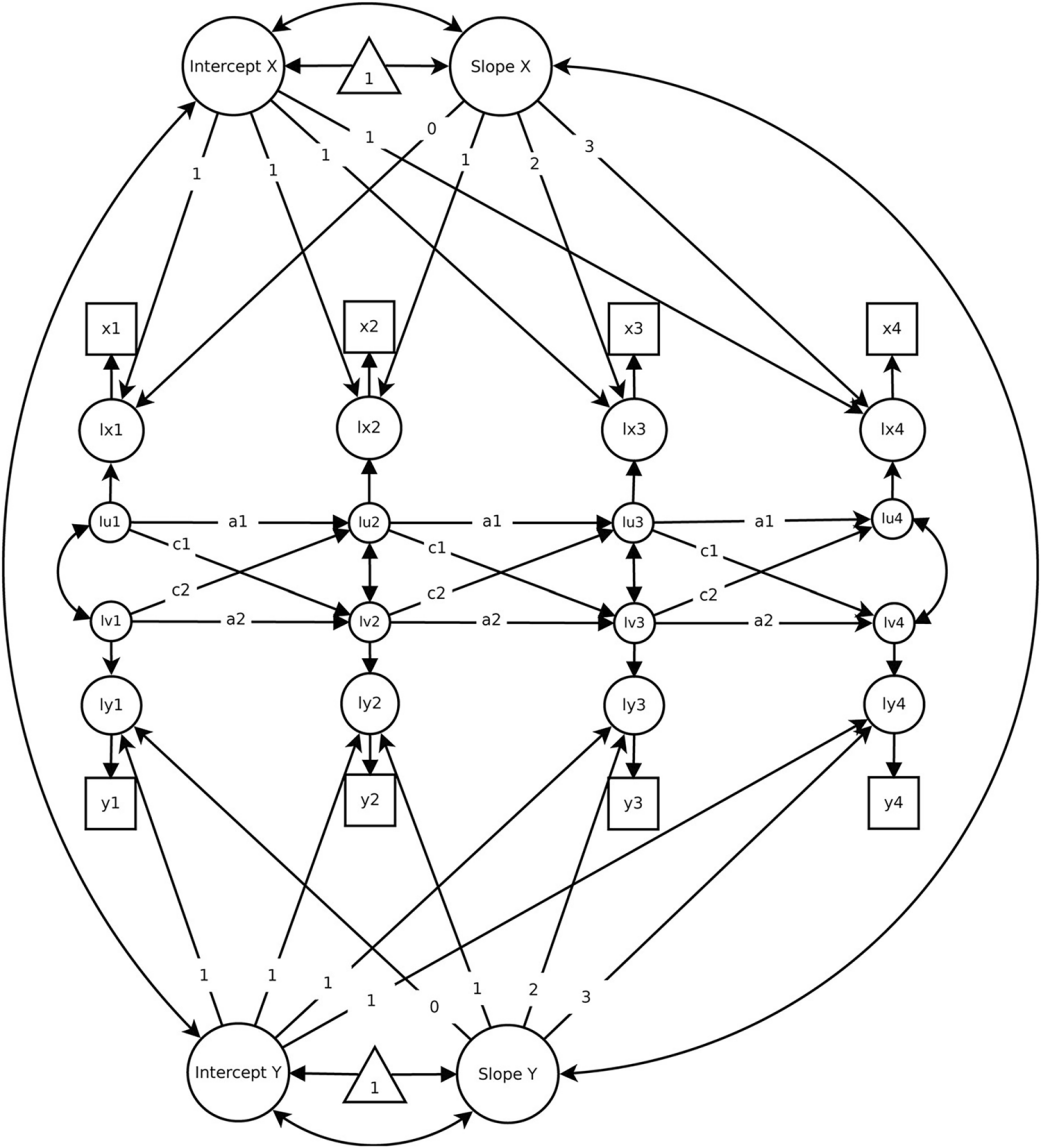
Autoregressive latent trajectory model with structured residuals (ALT-SR). Image from Mund and Nestler^16^ reproduced with permissions under a CC-BY 4.0 license. In the current study, time was coded as: 0, 1, 2, 3,…, 32.

Figure 1 depicts a basic (i.e., considering two variables measured across only four time points) ALT-SR model^16^. To test our hypotheses and to reduce computational load, our focal model was specified with all over-time parameters (i.e., autoregressive and cross-lagged effects) fixed to equality. We also tested a model that freed these assumptions (i.e., a model with freely-estimated autoregressive and cross-lagged effects). Finally, we considered a model with linear constraints imposed on cross-lagged effects (i.e., κ constraints; see^27^, formula 17), which allows us to test for systematic changes in the strength of such effects over time.

To address missing data, we considered a set of descriptive analyses comparing complete (i.e., participants in all 33 waves; *n* = 148) to incomplete (i.e., participants in waves T3 to T35; *n* = 1513) responders in our panel (see also Table 1). No statistically significant differences were observed between demographics and indicators of socioeconomic status for complete and incomplete responders. In a logistic regression model, demographics, indicators of socioeconomic status, and substantive variables (at T3) explained approximately 1% (*R*^2^ = 0.01) of the variance in response status. Complete results of this analysis can be found in our Supplemental Materials. In estimating our models, full information maximum likelihood (FIML) was used to account for observed missingness.

### Ethics approval

This study was approved by the ethics advisory board of Leipzig University (Protocol ID# 2019.06.27_eb_17, study title: Longitudinal study on experiences and behavior at work). The research was performed in accordance with the ethical standards and guidelines by the ethics advisory board of Leipzig University, national regulations by the German Science Foundation, and in accordance with the Declaration of Helsinki.

### Informed consent

Informed consent about participation and publication was obtained from all individual participants included in the study. Participation in the study was voluntary, and data were saved and processed anonymously.

## Results

As an initial step in modeling over-time change, we considered measurement invariance of digitalization and workload at three equidistant time points (i.e., T3, T19, T35) to reduce computational demands. Briefly, metric invariance was upheld across time for digitalization and workload; complete results of these models can be found in our Supplemental Materials. Descriptive statistics and correlations among substantive variables can also be found in our Supplemental Materials. There was a notable amount of within-person variability in digitalization and workload over time, as indexed by ICC_1_ statistics (i.e., 25% of the variability in digitalization and 32.50% of the variability in workload occurred within person, over time).

Table 2 summarizes the results of the ALT-SR model specified to test our hypotheses. This model fit the data well [χ^2^_(2191)_ = 4409.485, CFI = 0.973, TLI = 0.974, RMSEA = 0.025, 95% CI 0.024; 0.026, SRMR = 0.042]. There are multiple components to the ALT-SR model, described next, using established notation^27^. In terms of growth parameters, representing average between-person levels (i.e., intercepts; ⍺) and changes (i.e., slopes; β), we noted a statistically significant (*p* < 0.05) positive average trajectory of digitalization (β_dig_ = 0.003), but not of workload. This suggests that, on average, digitalization increased across time, in this sample.

**Table 2:** Summary of focal autoregressive latent trajectory model with structured residuals (ALT-SR) parameters.

Parameter type	Parameter	Est.	SE	Z	p	Lower	Upper	Std. est.
Autoregressive effects	ρ_dig→dig_	0.159	0.007	22.708	< 0.001	0.145	0.173	0.148
	ρ_wor→wor_	0.236	0.007	34.387	< 0.001	0.222	0.249	0.214
Cross-lagged effects	ρ_dig→wor_	0.025	0.006	4.003	< 0.001	0.013	0.038	0.023
	ρ_wor→dig_	0.035	0.007	5.238	< 0.001	0.022	0.048	0.033
Latent trajectories	⍺_dig_	4.628	0.036	128.901	< 0.001	4.557	4.698	3.317
	⍺_wor_	4.189	0.032	132.959	< 0.001	4.127	4.250	3.494
	β_dig_	0.003	0.001	2.613	0.009	0.001	0.005	0.087
	β_wor_	0.001	0.001	0.838	0.402	− 0.001	0.003	0.027
Variances of latent trajectories	σ^2^_⍺dig_	1.946	0.074	26.183	< 0.001	1.801	2.092	1.000
	σ^2^_⍺wor_	1.437	0.057	25.196	< 0.001	1.325	1.549	1.000
	σ^2^_βdig_	0.001	0.000	16.473	< 0.001	0.001	0.001	1.000
	σ^2^_βwor_	0.001	0.000	17.219	< 0.001	0.001	0.001	1.000
Correlations among latent trajectories	ψ_⍺dig ⍺wor_	0.166	0.027	6.224	< 0.001	0.114	0.219	0.166
	ψ_βdig βwor_	0.255	0.039	6.460	< 0.001	0.178	0.332	0.255
	ψ_⍺dig βdig_	− 0.079	0.034	− 2.303	0.021	− 0.146	− 0.012	− 0.079
	ψ_⍺dig βwor_	0.012	0.034	0.365	0.715	− 0.053	0.078	0.012
	ψ_⍺wor βdig_	− 0.079	0.035	− 2.292	0.022	− 0.147	− 0.012	− 0.079
	ψ_⍺wor βwor_	− 0.222	0.032	− 6.982	< 0.001	− 0.284	− 0.159	− 0.222

*dig* workplace digitalization, *work* workload, *ρ* autoregressive and cross-lagged parameters, *⍺* latent intercepts, *β* latent slopes, *σ*^2^ variances, *ψ* correlations.

We also noted a correlation (ψ) between slopes consistent with Hypotheses 1 and 3, representing a “change–change” relation that is dynamic (on average) over time. Specifically, the correlation between the slopes of workplace digitalization and workload (ψ_βdig βwor_ = 0.255) was significant and positive, suggesting that on-average changes in digitalization and on-average changes in workload are positively related.

In terms of the autoregressive effects (e.g., ρ_dig→dig_, corresponding to the pathways labeled “a” in Fig. 1), we noted significant and positive “stabilities” in both digitalization and workload (ρ_dig→dig_ = 0.159; ρ_wor→wor_ = 0.236).

In terms of the within-person cross-lagged effects (e.g., ρ_dig→wor_, ρ_wor→dig_, corresponding to the pathways labeled “c” in Fig. 1) that serve as the tests of our hypotheses, we noted that digitalization at T_*k*_ was significantly and positively associated with workload at T_*k*+1_ (ρ_dig→dem_ = 0.025). Thus, Hypothesis 1 was supported, whereas Hypothesis 2 was not supported. Moreover, in support of Hypothesis 3, workload at T_*k*_ was significantly and positively associated with digitalization at T_*k*+1_ (ρ_dem→dig_ = 0.035). In contrast, Hypothesis 4 did not receive support. Overall, these findings suggest that digitalization predicts subsequently higher levels of workload, and vice versa.

### Additional analyses

**Figure 2:**
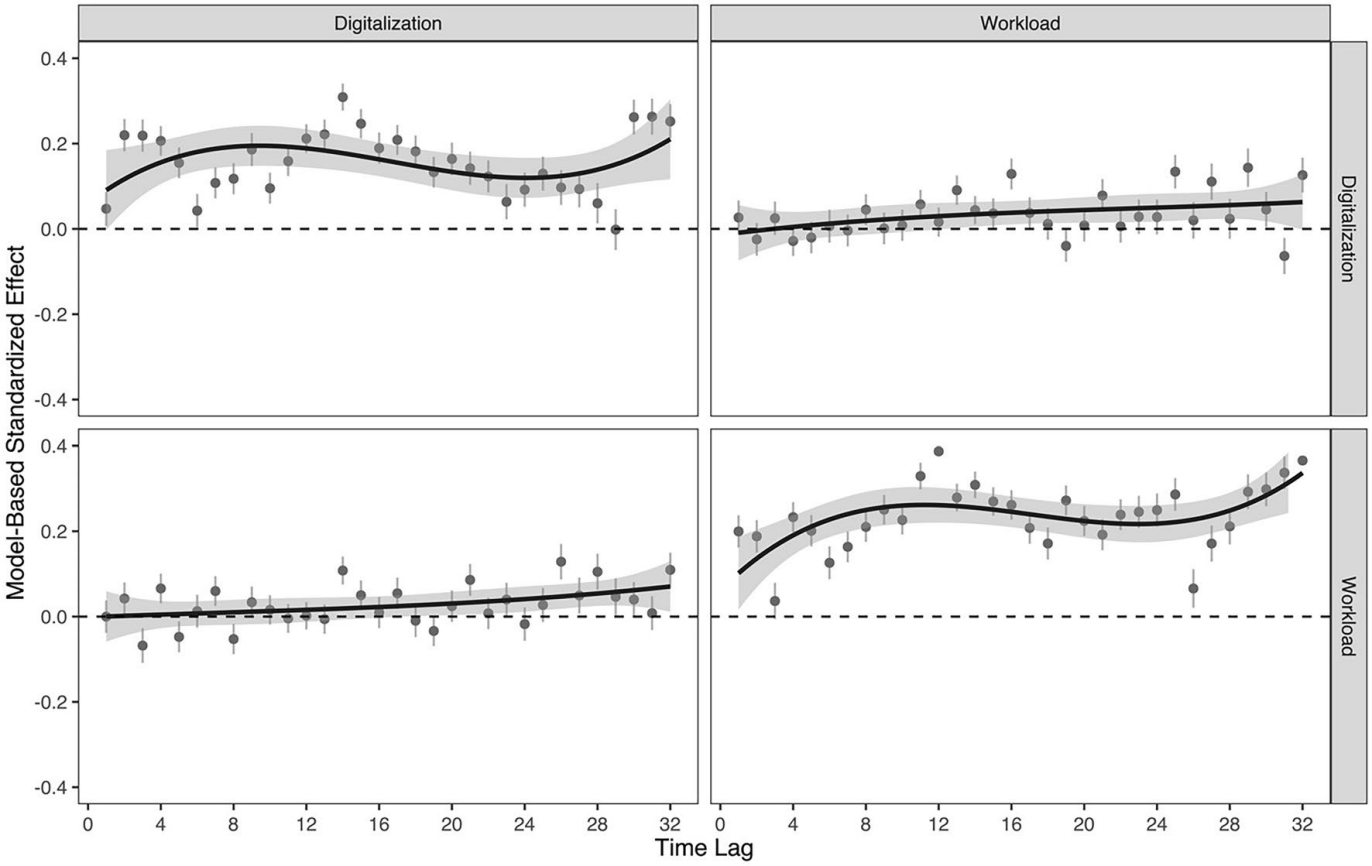
Fully unrestricted autoregressive and cross-lagged effects of workplace digitalization and workload. Plots along the diagonal represent autoregressive effects; off diagonal plots represent cross-lagged effects following [“to” = row, “from” = column] matrix logic (e.g., cross-lagged effects to workload from workplace digitalization are in row two, column one [2, 1]; cross-lagged effects to workplace digitalization from workload are in row one, column two [1, 2]. Standardized effects are depicted here to ensure consistent scaling across estimate parameters. Error bars/bands represent 95% confidence intervals around LOESS estimates and are presented for descriptive purposes only.

We further specified two competing ALT-SR models that freed over-time parameters in our focal model into fully unrestricted models. Specifically, we specified a fully unrestricted model, with all auto- and cross-lagged effects freely estimated. Complete results of these analyses can be found in our Supplemental Materials. Figure 2 displays plots that depict standardized autoregressive and cross-lagged effects of workplace digitalization and workload in this fully unrestricted model. Comparing the fits of these less-constrained models to our focal models resulted in absolute improvements in overall model fit (i.e., as indexed by chi-square difference tests); however, the relative fit of these models to the data (indexed by CFI, TLI, and RMSEA values) was equivalent between our focal and alternative models. Consistent with our theorizing and hypotheses, we favor the more parsimonious representation of these data, but present all specifications here in service of transparency and openness.

**Figure 3:**
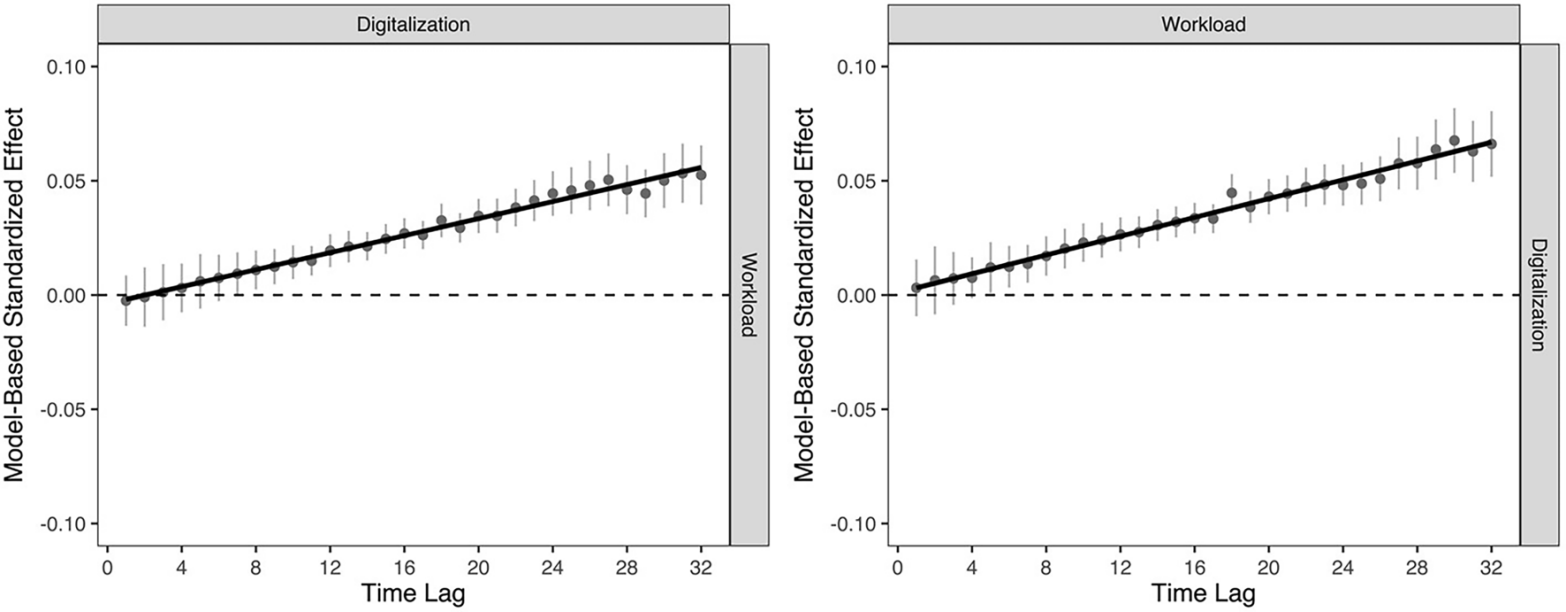
Positive linear trend (i.e., κ) in the within-person relations from workplace digitalization to workload (**A**) and workload to workplace digitalization (**B**). Standardized effects are depicted here to ensure consistent scaling across estimated parameters. Error bars represent 95% confidence intervals for each cross-lagged parameter. GLM estimates slopes are presented here for descriptive purposes only.

From this specification, we noted that there are slight linear trends in the patterns of cross-lagged effects between digitalization and workload, and vice versa (see Fig. 2). To formally test whether these trends are statistically significant, we respecified this model with linear constraints imposed on the cross-lagged effects (i.e., κ constraints; see^27^, formula 17) to test for systematic changes in such effects over time. We found significant and positive linear trends in the within-person association between digitalization and workload, ρ_dig→wor_ (i.e., κ _dig→dem_ = 0.002) and in the within-person association between workload and digitalization, ρ_wor→dig_ (i.e., κ _dem→dig_ = 0.002), suggesting that the strength of these lagged associations becomes more strongly positive across the study period (see Fig. 3).

## Discussion

Both workplace digitalization and work design for improved employee health and effectiveness are two important trends in organizational research and practice^2,9^. Based on theorizing on workplace ICT use^6^, the first goal of this study was to examine the effect of digitalization on workload at the within-person level of analysis. Research concerning this association, which has mostly relied on cross-sectional, between-person research designs, has suggested that digitalization could lead to both an increase (i.e., due to information processing demands and overload, new learning and adaptation requirements, and ICT-related problems) or a decrease (i.e., due to a reduction in cognitive effort required, and by streamlining work processes to make them more effective) in workload. Consistent with the former possibility, we found support for a positive within-person effect of digitalization on workload, suggesting that the digitalization at work may create new demands, requirements, and problems. Our findings are consistent with results of both qualitative and quantitative research on relations between digitalization and workload. For example, a qualitative study based on 16 interviews with knowledge workers from the medical, clergy, legal, and teaching professions, suggested that digitalization can lead to an increased workload, particularly due to higher mental demands^19^. Moreover, a quantitative study based on archival data examined how digitalization impacted workload in preservation departments of 18 libraries over the course of 5 years^30^. Results suggested that the number of items processed by library staff increased by 10% due to digital reformatting tasks, without a corresponding increase in staffing.

The second goal of our study was to expand theorizing on workplace ICT use by considering the reverse effect of workload on digitalization at the within-person level, which has so far been neglected in the theoretical and empirical literature on digitalization and work design^6^. Based on theorizing on stress and coping^15^, we argued that, on the one hand, a high workload could be appraised as a challenge and opportunity to cope. This appraisal, in turn, should result in increased digitalization as a means of managing a high workload. On the other hand, we suggested that a high workload could also be appraised as a threat that offers limited opportunities to cope in a problem-oriented way and, thus, should reduce digitalization over time. Consistent with the former argument, we found that workload leads to increases in digitalization, suggesting that ICT use may indeed represent a way of coping with the challenges associated with a high workload.

Our findings of positive reciprocal relations between workplace digitalization and workload over time may suggest certain “spiraling effects”, wherein positive deviations from an individual’s average trajectory of digitalization are associated with subsequently higher levels of workload, and vice versa. On the one hand, the finding that digitalization leads to subsequent increases in workload, which in turn further increases digitalization, may suggest a “loss spiral”^31^. That is, digitalization leads to new information processing demands, learning and adapting requirements, and ICT problems—each of which can increase workload and, thus, consume personal resources, such as mental and emotional energy. On the other hand, however, the finding that workload also leads to increases in digitalization over time could be interpreted as a “gain spiral”^31^, as digitalization may not only increase workload, but could also help make certain work processes more efficient and, thus, reduce one’s need to invest additional cognitive effort to accomplish one’s work tasks.

In contrast to most previous research in this area, we used a longitudinal research design with 33 monthly measurement waves across nearly three years and a statistical modeling strategy (ALT-SR) that allows for robust and simultaneous estimation of dynamic and reciprocal within-person processes. This approach also allowed us to address a key limitation of previous research, which has confounded digitalization and workload, for instance by investigating composite constructs that confound digitalization with workload (e.g., “ICT demands”^10^). Our additional analyses revealed that there was an on-average positive linear trajectory in workplace digitalization, but not in workload, over time. The former finding may be explained by the timing of our study, which started at the advent of the COVID-19 pandemic at the beginning of April 2020 in Germany, which accelerated many already present features of workplace digitalization (e.g., working from home, virtual team meetings^8^). Interestingly, the overall increase in digitalization was not matched by a simultaneous overall increase in workload across the same time period. Research on working during the COVID-19 pandemic suggests that certain changes may have led to a temporary increase in workload (e.g., requirements to follow hygiene rules), whereas other changes may have reduced employees’ workload (e.g., fewer customers^8^). Moreover, employees have been shown to adapt to work-related changes during the pandemic that may threaten their job performance^32^. Overall, these divergent changes and mechanisms might have resulted in a rather stable workload over time.

We additionally found evidence for linear trends in the identified within-person processes themselves, suggesting that the strength of the within-person effects of digitalization on workload, and of workload on digitalization, becomes more strongly positive over time. A potential explanation for this exploratory result may be that, throughout the COVID-19 pandemic and beyond, the perceived amount and difficulty of work (i.e., in terms of the volume and pace of the work required of employees; their work demands) became increasingly linked with individual, team, and organizational efforts to digitalize various work processes^33,34^. Overall, however, our findings suggest that the relation between workplace digitalization and workload is complex, reciprocal, and intensifying over time.

### Theoretical and practical implications

Our findings have implications for theorizing on the antecedents and consequences of both workplace digitalization^2,6^ and workload^14,18^. First, based on our findings, theorizing on workplace ICT use can be extended by explicitly considering both between- and within-person levels of analysis. Whereas existing evidence at the between-person level of analysis seems rather mixed, our findings at the within-person level of analysis suggest a generally positive effect of digitalization on workload. Moreover, models of workplace ICT use^6^ should be expanded to include the reverse effect of workload (and potentially other work characteristics) on digitalization and ICT use at the workplace. As demonstrated in the present study, employees’ workload may influence the implementation and use of digital technologies in the workplace, which should be reflected in revised theoretical models and future research. Second, theorizing and empirical research on workload has focused on associations with other work characteristics (e.g., social support) and employee outcomes (e.g., strain), but has generally neglected workplace digitalization. Our findings suggest that digitalization, referring both to requirements to use and the dependency on ICT, should be formally integrated into more general models of work design, such as the job demands-resources model^35^. Importantly, our findings suggest a need for such models to consider digitalization both as a job demand and a resource.

In terms of practical implications, organizations may leverage our findings to balance the benefits and challenges of digitalization. First, organizational consultants and practitioners could use our findings to inform and develop interventions that address ways to reduce the detrimental impact of digitalization on increased workload. For example, it seems important that increases in digitalization are accompanied by employee training to develop “digital competencies”, including the effective use of technology^36^. Moreover, work design interventions could be used to increase other work characteristics, such as job autonomy, team cohesion, and task significance^37^, which may buffer the effects of digitalization on increases in workload. At the same time, given the potential benefits of increased digitalization apart from an increased workload, interventions could consider how to best facilitate digitalization efforts to help employees enact problem-focused coping and address high levels of workload. In this regard, organizational practitioners should explain both the potential opportunities (e.g., efficient and fast knowledge flow, independence, co-creation) and risks (e.g., information overload, “always-on mode”, time management challenges) of digitalization to employees and teach them how to best maximize the opportunities and minimize the risks^19^.

Our findings may also have several practical implications for post-pandemic work environments. Digitalization and related developments (e.g., use of artificial intelligence) will likely further increase in workplaces in the coming years and have been identified as the primary drivers of the “future of work”^38^. Employees, supervisors, and human resource management practitioners should not only focus on the potential benefits of digitalization, but also be aware of its potential risks. According to our findings, a key risk of increased digitalization is increased workload, which is associated with reduced employee health and wellbeing^13^. To attract and retain staff in the future, organizations need to find ways to make effective use of digitalization without increasing workload. Indeed, our findings suggest the possibility that the effective use of digitalization in organizations may be a strategy to cope with a high workload.

### Limitations and directions for future research

This study has several strengths, including the focus on relatively long-term and reciprocal relations between workplace digitalization and workload across time. Despite this, our study also has some limitations that could be addressed in future research. First, we did not conduct a randomized-controlled experiment in which digitalization and workload were systematically manipulated. Thus, we are not able to make strong conclusions about causality^39^. However, our longitudinal research design with 33 measurement waves and the use of advanced statistical techniques that control for general trends and autoregressive effects allow us to determine the temporal precedence of variables that explain subsequent changes in other variables over time.

Second, both digitalization and workload were self-reported by employees, and self-reports are prone to various biases that might inflate statistical estimates (e.g., self-enhancement, common method variance). For example, at the between-person level, a cross-sectional estimate of the correlation between digitalization and workload may be artificially inflated because self-reports of workplace characteristics may be confounded by individual differences, such as the stable tendencies to experience positive or negative affect^40^. If such confounds are not controlled, the association between two self-reported constructs may appear larger than it truly is. Our consideration of longitudinal, within-person data, with controls for both average trends and autoregressive effects, should assuage such concerns to some extent. However, future studies could additionally explore effects of more objective assessments of both workplace digitalization and workload (e.g., archival records, observational data, supervisor and coworker ratings).

Third, workload has been conceptualized as a multidimensional construct, including both quantitative and qualitative workload dimensions. However, most studies, including a meta-analysis on workload^18^, do not distinguish these two dimensions due to a lack of systematic research on their differences. In our study, consistent with most previous research, we focused on quantitative workload (i.e., having too much to do in too little time^13^). Thus, our findings may not generalize to qualitative workload, which entails having too many complex and difficult tasks to do in one’s available time at work (a related work characteristic is called “job complexity”^41^). Future research should include distinct measures of both qualitative and quantitative workload and examine their reciprocal associations with workplace digitalization.

Fourth, our longitudinal study was carried out during the COVID-19 pandemic, which started in mid-March 2020 and was declared “over” by government officials in Germany in the spring of 2023. Accordingly, our study design does now allow us to directly compare our findings to associations among workplace digitalization and workload before or after the pandemic. It is likely that certain responses to the pandemic (e.g., rapid reorganization of work from in-person to virtual) has led to greater between- and within-person variability in the focal study variables, compared to what might be observed pre- or post-pandemic^42^. Future research conducted in a post-pandemic context could attempt to replicate our findings, which may also be interesting to study in situ, for example, in the context of new ICT implementations rolled out over time within a single organization. Because such implementations are often done in a piecemeal fashion (e.g., implementations are introduced division-by-division), they represent an interesting opportunity to consider a “natural experiment” of associations between digitalization and workload.

Finally, and related to the previous point, we cannot explain *why* systematic changes in the strength of reciprocal within-person associations between digitalization and workload occurred over time, other than attributing these changes to various developments in workplaces associated with the dynamic pandemic context. For example, systematic changes may reflect developmental effects (i.e., dynamics associated with the normal passing of time) or the cumulative influence of exogenous “shocks”^42^. Future research could address these possible explanations, and consider whether contextual factors (e.g., employee’s readiness for technology implementation; employees’ acceptance of ICT) help explain or modify these processes.

## Conclusion

In this study, we focused on two constructs that reflect broader trends in society and organizations: digitalization and workload. We contribute to the literature by presenting the results a unique longitudinal study with 33 monthly measurement points across nearly three years and a large sample of employees in Germany. We advance understanding of the digital transformation of workplaces by measuring workplace digitalization and workload as distinct constructs and analyzing their potential reciprocal effects over time. We found positive, within-person reciprocal effects between digitalization and workload. Specifically, higher workplace digitalization led to increases in workload over time, and higher workload led to increases in digitalization over time. Digitalization, but not workload, increased linearly across the study period (during the COVID-19 pandemic). Finally, the positive and reciprocal within-person relations between digitalization and workload became stronger over time. These findings suggest that the relation between digitalization and workload is complex, dynamic, and reciprocal.

### Supplementary Information

Supplementary Table S1.

## Data Availability

Data, R code to replicate the analyses, and complete results are available in our Supplemental Materials: https://osf.io/5gebw/.
