# Supplementary material for: Workplace digitalization and workload: changes and reciprocal relations across 3 years
Source: Sci Rep. 2024 Mar 11;14:5924. doi: 10.1038/s41598-024-56537-w (PMC10928114; doi:10.1038/s41598-024-56537-w)
Supplement: Supplementary file 1 — Supplementary Table S1. [file 41598_2024_56537_MOESM1_ESM.pdf]

Table S1

Published Studies Based on Longitudinal Dataset

Study	Measurement Waves Used	Substantive Variables Used
1. Zacher and Rudolph (2021b)	T1-T4	Life satisfaction, positive affect, negative affect, stress appraisals, coping strategies
2. Zacher and Rudolph (2021a)	T1, T3, T4, T6-T8	Perceived stressfulness of the COVID-19 pandemic, Big Five personality traits (only T1)
3. Rudolph and Zacher (2021)	T1, T3, T4, T6-T8	Family demands, satisfaction with family life, partnership status, parental status, job demands
4. Zacher et al. (2021)	T1-T8	Work performance (task proficiency, adaptivity, proactivity), core self-evaluations, work-related changes due to the lockdown
5. Koziel et al. (2021)	T1, T2, T5	Age-differentiated leadership, leader-member exchange, work ability
6. Rudolph et al. (2022)	T1, T2, T5	Directive and empowering leadership, emotional engagement, emotional fatigue
7. Rauvola et al. (2022)	T1-T17	Work fatigue (physical, mental, emotional), short-term work
8. M. Weiss et al. (2022)	T1, T2, T5	Essentialist beliefs, age stereotypes, occupational future time perspective, motivation to continue working
9. D. Weiss et al. (2022)	T1-T7	Occupational status, perceived constraints at work, job satisfaction
10. Rudolph and Zacher (2023)	T1, T5, T14, T18, T20, T22-T24, T29, T33, T35	Big Five personality traits, physical and mental health (only T1 & T22)
11. Zacher and Rudolph (2023)	T1-T35	Life satisfaction, positive affect, negative affect, stress appraisals, coping strategies
12. Zacher (2024)	T35 & T36	Dark triad traits, left- and right-wing authoritarianism, environmental activism, Big Five personality traits
**13. Current Study**	**T1, T3-T35**	**Workplace digitalization, workload**

References

Koziel, R., Friedrich, J. C., Rudolph, C. W., & Zacher, H. (2021). Age-differentiated leadership and healthy aging at work: Evidence from the early stages of the COVID-19 pandemic. *International Journal of Environmental Research and Public Health*, *18*(23), 12509. https://doi.org/10.3390/ijerph182312509

Rauvola, R. S., Rudolph, C. W., & Zacher, H. (2022). Short-term effects of short-term work: Dynamics in fatigue across two national lockdowns. *Journal of Occupational and Environmental Medicine*, *64*(7), 550-556. https://doi.org/10.1097/JOM.0000000000002537

Rudolph, C. W., Breevaart, K., & Zacher, H. (2022). Disentangling between-person and reciprocal within-person relationships between perceived leadership and employee wellbeing. *Journal of Occupational Health Psychology*, *27*(4), 441–450. https://doi.org/10.1037/ocp0000320

Rudolph, C. W., & Zacher, H. (2021). Family demands and satisfaction with family life during the COVID-19 pandemic. *Couple and Family Psychology: Research and Practice*, *10*(4), 249-259. https://doi.org/10.1037/cfp0000170

Rudolph, C. W., & Zacher, H. (2023). Individual differences and changes in personality during the COVID-19 pandemic. *Social and Personality Psychology Compass*, *17*(7), e12742. https://doi.org/10.1111/spc3.12742

Weiss, D., Weiss, M., Rudolph, C. W., & Zacher, H. (2022). Tough times at the top: Occupational status predicts changes in job satisfaction in times of crisis. *Journal of Vocational Behavior*, *139*(1), 103804. https://doi.org/10.1016/j.jvb.2022.103804

Weiss, M., Weiss, D., & Zacher, H. (2022). All set in stone? How and why essentialist beliefs about aging affect employees' motivation to continue working beyond retirement age. *Journal of Organizational Behavior*, *43*(8), 1446-1461. https://doi.org/10.1002/job.2647

Zacher, H. (2024). The dark side of environmental activism. *Personality and Individual Differences*, *219*(1), 112506. https://doi.org/10.1016/j.paid.2023.112506

Zacher, H., & Rudolph, C. W. (2021a). Big Five Traits as predictors of perceived stressfulness of the COVID-19 pandemic. *Personality and Individual Differences*, *175*, 110694. https://doi.org/10.1016/j.paid.2021.110694

Zacher, H., & Rudolph, C. W. (2021b). Individual differences and changes in subjective wellbeing during the early stages of the COVID-19 pandemic. *American Psychologist*, *76*(1), 50-62. https://doi.org/10.1037/amp0000702

Zacher, H., & Rudolph, C. W. (2023). Subjective wellbeing during the COVID-19 pandemic: A 3-year, 35-wave longitudinal study. *The Journal of Positive Psychology*. https://doi.org/10.1080/17439760.2023.2224757

Zacher, H., Rudolph, C. W., & Posch, M. (2021). Individual differences and changes in self-reported work performance during the early stages of the COVID-19 pandemic. *Zeitschrift für Arbeits- und Organisationspsychologie*, *65*(4). https://doi.org/10.026/0932-4089/a000365
